# Identification of Zinc-Binding Inhibitors of Matrix Metalloproteinase-9 to Prevent Cancer Through Deep Learning and Molecular Dynamics Simulation Approach

**DOI:** 10.3389/fmolb.2022.857430

**Published:** 2022-03-31

**Authors:** Shalini Mathpal, Priyanka Sharma, Tushar Joshi, Veena Pande, Shafi Mahmud, Mi-Kyung Jeong, Ahmad J. Obaidullah, Subhash Chandra, Bonglee Kim

**Affiliations:** ^1^ Department of Biotechnology, Kumaun University Uttarakhand, Bhimtal, India; ^2^ Department of Botany, DSB, Campus, Kumaun University, Nainital, India; ^3^ Genetic Engineering and Biotechnology, University of Rajshahi, Rajshahi, Bangladesh; ^4^ Department of Genome Science, John Curtin School of Medical Research, Australian National University, Canberra, Australia; ^5^ KM Convergence Research Division, Korea Institute of Oriental Medicine, Daejeon, South Korea; ^6^ Drug Exploration and Development Chair (DEDC), Department of Pharmaceutical Chemistry, College of Pharmacy, King Saud University, Riyadh, Saudi Arabia; ^7^ Computational Biology and Biotechnology Laboratory, Department of Botany, Soban Singh Jeena University, Almora, India; ^8^ Department of Pathology, College of Korean Medicine, Kyung Hee University, Seoul, South Korea

**Keywords:** cancer, MMP-9, deep learning, drug bank compounds, MD simulation

## Abstract

The overexpression of matrix metalloproteinase-9 (MMP-9) is associated with tumor development and angiogenesis, and hence, it has been considered an attractive drug target for anticancer therapy. To assist in drug design endeavors for MMP-9 targets, an *in silico* study was presented to investigate whether our compounds inhibit MMP-9 by binding to the catalytic domain, similar to their inhibitor or not. For that, in the initial stage, a deep-learning algorithm was used for the predictive modeling of the CHEMBL321 dataset of MMP-9 inhibitors. Several regression models were built and evaluated based on R2, MAE MSE, RMSE, and Loss. The best model was utilized to screen the drug bank database containing 9,102 compounds to seek novel compounds as MMP-9 inhibitors. Then top high score compounds were selected for molecular docking based on the comparison between the score of the reference molecule. Furthermore, molecules having the highest docking scores were selected, and interaction mechanisms with respect to S1 pocket and catalytic zinc ion of these compounds were also discussed. Those compounds, involving binding to the catalytic zinc ion and the S1 pocket of MMP-9, were considered preferentially for molecular dynamics studies (100 ns) and an MM-PBSA (last 30 ns) analysis. Based on the results, we proposed several novel compounds as potential candidates for MMP-9 inhibition and investigated their binding properties with MMP-9. The findings suggested that these compounds may be useful in the design and development of MMP-9 inhibitors in the future.

## Introduction

MMPs (matrix metalloproteinases) are zinc-dependent endopeptidases with more than 20 different members (Klein and Bischoff, 2011). MMPs can be divided into six main groups, such as gelatinases, collagenases, matrilysins, stromelysins, membrane-type MMPs, and others (Nagase et al., 2006). In particular, MMP-9, a member of the gelatinase family, is one of the most complex action-performing MMPs, as it plays a key role in cancer cell invasion, tumor metastasis, and triggering the “angiogenic switch” (Bronisz and Kurkowska-Jastrzębska, 2016). During tissue remodeling, it degrades gelatin and types IV, V, XI, and XVI collagen, which are essential for tumor invasion and metastasis (Liu et al., 2015). Additionally, MMP-9 also acts as a functional component of angiogenesis switch during multistage carcinogenesis, where MMP-9 triggers the release of a vascular epidermal growth factor, which accelerates tumor growth (Engsig et al., 2000). MMP-9 is commonly found in the hippocampus, cerebellum, and cerebral cortex. It is secreted mainly from the endothelial cells, leukocytes, fibroblasts, neutrophils, and macrophages as zymogens or in an inactive form (Bronisz and Kurkowska-Jastrzębska, 2016).

Human MMP-9 proteins generally consist of three domains: the N-terminal propeptide domain, the catalytic domain, and the C-terminal, the hemopexin-like domain. The catalytic domain of human MMP-9 which is without the fibronectin repeats has the same structure as other MMPs: a five-stranded beta-sheet and three alpha-helices. It is composed of the active-site zinc ion, coordinated by three histidine residues (401, 405, and 411) and the essential glutamic acid residue (402). The catalytic zinc ion is essential for proteolytic activity (Bode et al., 1999).

The hemopexin-like domain of MMP-9 interacts with substrates like gelatin and collagen. This domain is crucial for substrate recognition (Roderfeld et al., 2007). Some natural tissue inhibitors of MMPs (TIMPs), such as TIMP-1, can bind to the hemopexin-like domain of pro-MMP-9 to form a tight complex that prevents MMP-9 from being activated (Nagase et al., 2006; Roderfeld et al., 2007).

Finding the drugs which can control the activity of MMP-9 is an important area of cancer research. MMP-9 overexpression has been observed in a variety of malignant cancers and has been extensively studied as a potential biomarker for several cancers. MMP-9 is found to play a crucial role in gastric cancer, breast cancer, colorectal cancer, non-small-cell lung cancer, ovarian cancer, and many other cancers (Roy et al., 2009; Shao et al., 2011; Hu et al., 2012; Li et al., 2013; Liang and Chang, 2018). Because of the important role of MMP-9 in tumorigenesis, it has been postulated to be a good prognostic marker and has been considered an attractive target in cancer therapy (Duffy, 1996). As a result, various MMP-9 synthetic inhibitors have been developed and used in clinical trials to prevent tumor metastasis, but the results have been unsatisfactory because of high toxicity and the lack of specificity (Coussens, 2002). In recent years, various natural and synthetic inhibitors of the enzyme MMP-9 have been synthesized and reported to be beneficial in the treatment of various cancerous conditions (Singh et al., 2017; Rathee et al., 2018, Rathee et al., 2019; Bursal et al., 2019; Lou et al., 2019; Phytochemical, 2020; Paramashivam and Narayanan, 2021).

**FIGURE 1:**
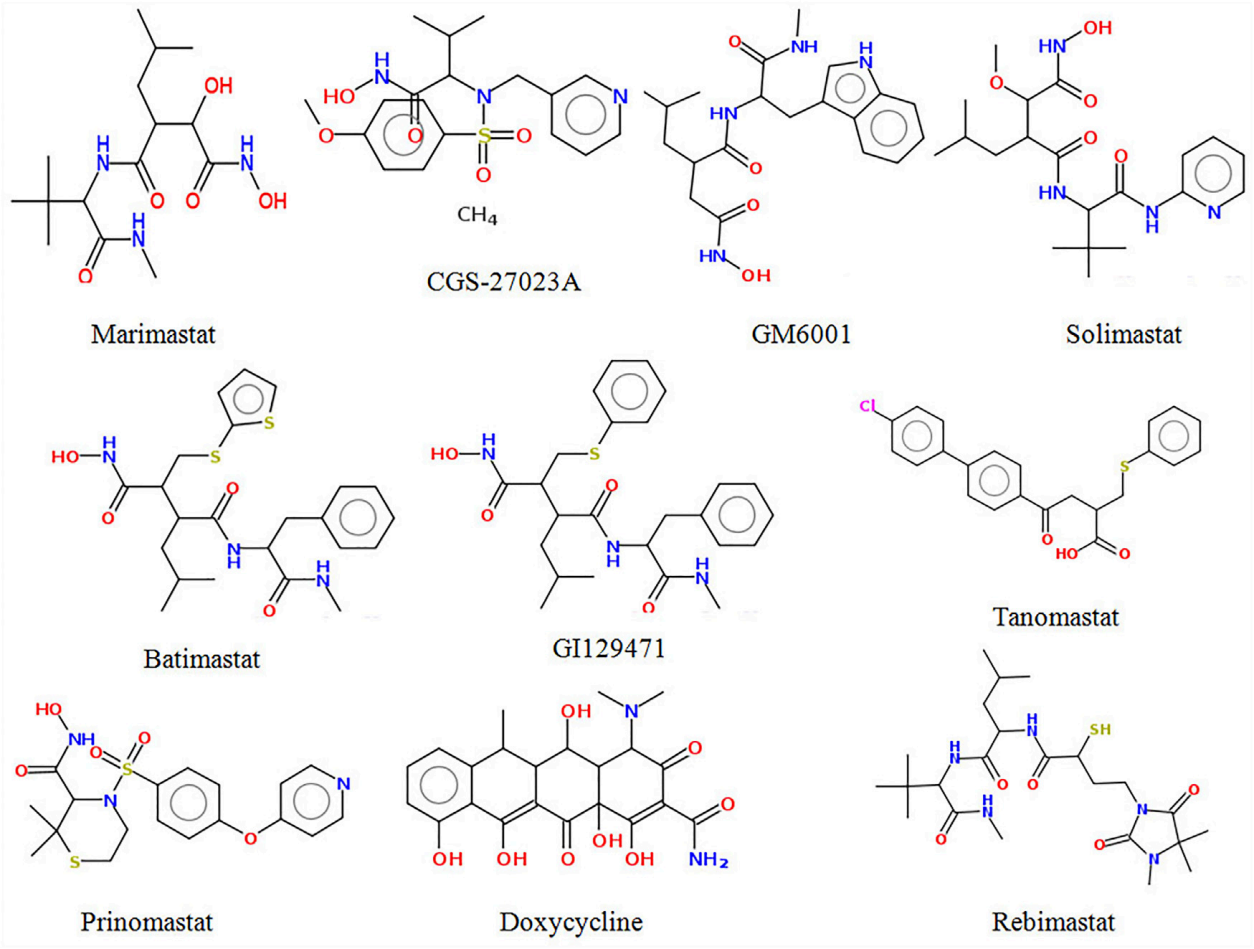
Structure of some potent MMP-9 inhibitors.

It has been suggested that molecules with a high affinity for Zn^2+^ effectively prevent the polypeptide from binding to MMPs, and are therefore considered MMP inhibitors (Tu et al., 2008). Zn^2+^ binding groups have been reported in several studies, including hydroxamic acid, thiols, carboxylates, and phosphonic acid, of which hydroxamic acid appears to be the most potent one among them (Whittaker et al., 1999; Skiles et al., 2001). Small MMP inhibitors with a broad spectrum of ZBG-containing activity have also been tested in cancer clinical trials by several pharmaceutical companies (Fisher and Mobashery, 2006; Fingleton, 2007). These include hydroxamate-based MMP inhibitors such as Marimastat, Batimastat, Solimastat, MMI-270, Trocade, Periomastat, and Tanomastat (Figure 1). However, almost all of the aforementioned MMPIs failed clinical trials because of poor solubility, low oral bioavailability, and numerous side effects (Jacobsen et al., 2010; Winer et al., 2018).

Addressing these issues, the present study aimed to screen novel MMP-9 inhibitors with which to target the zinc and catalytic pockets of MMP-9 by using deep learning and other computational techniques. Deep learning is a machine learning technique that employs advanced algorithms based on artificial neural networks inspired by biological brain operations. It has multiple processing layers and artificial neurons to mimic the function of the human brain (Rusk, 2016). Deep learning is effective in a variety of areas, including image processing, self-driving cars, natural language processing, medical diagnosis, and drug development (Esteva et al., 2019).

Therefore, in this study, we combined deep learning, molecular docking, and a molecular dynamics approach to find key interactions between the protein and ligands that are accountable for the inhibitory activity of MMP-9. We also performed a post facto analysis of the MD trajectories of the highest active molecule to determine the binding free energies associated with protein–ligand complexation.

## Material and Methods

### Predictive Modeling by Deep Learning

In this study, a deep learning algorithm was used to develop a predictive model for screening novel compounds against MMP-9. The Deep Learning online server (http://deepscreening.xielab.net) (Liu et al., 2019) was used to generate this model. The predictive model was built using the CHEMBL321 dataset, which provided the IC50 value for inhibiting the MMP-9 activity.

The preprocessing of this CHEMBL dataset for molecular vectorization was performed by applying the PubChem Fingerprint which generates 881 fingerprints using PaDEL software (Yap, 2011).

The PubChem fingerprints were used to build regression models by applying deep recurrent neural networks (RNNs). To choose the best model, hyperparameters such as learning rate, epochs, batch size, number of neurons, and hidden layers were manually optimized. The ReLU (*y* = max (0,1)) activation function was used for the hidden layers, whereas the sigmoid function was used for the output layer.

### Model Evaluation and Virtual Screening

The deep learning models and the performance of the models were evaluated by various statistical parameters such as R-squared (R^2^), mean squared error (MSE), root MSE (RMSE), and mean absolute error (MAE).

The best regression model was deployed in the drug bank library (Library id-L00005) which contains 9,102 compounds for virtual screening, and the model predicted 500 screened hits.

$$MSE=\frac{1}{N}\overset{N}{\underset{i=1}{\sum }}{\left(y_{i}-\hat{y}\right)}^{2},$$ (1)

$$RMSE=\sqrt{MSE}=\sqrt{\frac{1}{N}}\overset{N}{\underset{i=1}{\sum }}{\left(y_{i}-\hat{y}\right)}^{2},$$ (2)

$$MAE=\frac{1}{N}\overset{N}{\underset{i=1}{\sum }}\left|y_{i}-\hat{y}\right|,$$ (3)

$$R^{2}=1-[\frac{\sum {\left(y_{i}-\hat{y}\right)}^{2}}{\sum \left(y_{i}-\bar{y}\right)}],$$ (4)

where y_*i*_ = observed value, $\hat{y}$ = predicted value, and $\bar{y}$ = mean value.

### Protein and Ligand Preparation

The 3D crystal structure of MMP-9 in a complex with a reverse hydroxamate inhibitor obtained from the protein data bank was used for this study. The PDB ID: 1GKC was chosen from 14 crystal structures because it is the first reported structure for MMP-9 with a reverse hydroxamate inhibitor. Reverse hydroxamate inhibitors have better pharmacokinetic properties than their hydroxamate counterparts, making them a better starting point for drug development (Rowsell et al., 2002). The binding site of MMP-9 (PDB ID: 1GKC) was then evaluated by PDBsum. The structure was optimized by adding hydrogen using the MGL Tools of AutoDockVina software. Water molecules, chain B, reverse hydroxamate inhibitors, and calcium ions were detached from the structure, and 3D protonation was conducted to convert the state into the ionization level.

A co-crystalized ligand as a reference molecule N∼2∼-[(2R)-2-{[formyl (hydroxy)amino]methyl}-4-methylpentanoyl]-N,3-dimethyl-L-valinamide or NFH (CID: 5287851) was downloaded from the PubChem server in the SDF format. All the selected ligands screened by deep learning and the reference molecule were converted to the PDB format by OpenBabel (version 2.3.1) (O’Boyle et al., 2011).

For the preparation of the ligands, hydrogen molecules were added to all hit compounds, and energy minimization was done with a UFF force field using an algorithm conjugated by PyRx software. All the compounds were later converted to the pdbqt format.

### Molecular Docking

The top hits shortlisted from the deep-learning screening were docked against MMP-9 using AutodockVina (Trott and Olson, 2009) to find the selective ligands for MMP-9.

The protein’s active site was generated using the default parameters of PyMOL software. The docking site on the protein target was determined by establishing a grid box with the dimensions of X: 25 Y: 25 Z: 25 Å, with a grid spacing of 0.375 Å, centered on X = 65.714, Y = 30.223, and Z = 117.65. Throughout the molecular docking, the receptor was set as a rigid body, whereas the ligands were kept flexible. To predict accurate results, the exhaustiveness default value was set to 8. The accuracy of the docking setup was validated by removing the reverse hydroxamate inhibitor from the catalytic domain of MMP-9 and re-docking with MMP-9 using the same grid and docking parameters as before. Finally, the ligands with the best confirmation and lowest binding free energy (G) were carefully chosen. The 2D and 3D interactions including hydrophobic, hydrogen bonds were further analyzed for the docked ligands by using Ligplotþv.1.4.5 (Wallace et al., 1995) and PyMOL software, respectively.

### Molecular Dynamics Simulation

MD simulations of 100 ns for all the systems were performed using GROMACS 5.0 package (Pronk et al., 2013). Energy optimization and calculations for protein and all the complexes were carried out by using the CHARMm27 force field. The structural topology files of all the complexes were generated using SwissParam (Zoete et al., 2011) web server. The initial complex was solvated in a pre-equilibrated orthorhombic box of TIP3P water (Jorgensen et al., 1983) containing box vectors of equal length, 9.81 nm, with dodecahedral periodic boundary conditions.

To neutralize the system, water molecules were added with sodium (Na^+^) and chloride (Cl^−^) ions, which directed periodic boundary conditions. Energy minimization using the steepest descent algorithm at 10 KJ/mol using the Verlet cutoff scheme taking the particle mesh Ewald (PME) columbic interactions and the total number of steps taken by all the systems during energy minimization cycle was 50,000. In the next phase, NVT and NPT equilibration was performed at a constant temperature of 300 K and 1.0 atm pressure with a time step of 2 fs, using the Parrinello-Rahman method for constant pressure simulation. According to Duan et al. (2019), 300 K is the standard and most appropriate temperature for MD simulations as it has the highest cluster occupancy (cluster analysis), lowest free energy state (free energy landscape analysis), and RMSD distribution. After the completion of 100 ns MD simulations of all the complexes, the post-processing and analyses of the MD trajectories were performed using analysis tools.

The tools of GROMACS 5.0.7 software package such as g_rms, g_rmsf, and g_gyrate, g_sasa, were used to analyze the root mean square deviation (RMSD), fluctuation (RMSF), radius of gyration (Rg), and solvent accessible surface area (SASA) of the MD trajectories. The number of hydrogen bonds formed within the complex and the protein during the MD simulation was calculated by hydrogen bond analysis.

### Molecular Mechanics Poisson–Boltzmann Surface Area Calculation

The binding free energy of top hit protein–ligand complexes was calculated using the MM-PBSA. This method directly estimates the free energies of end states by eliminating the simulation of intermediate states and then combines molecular mechanical energies with continuum solvent strategies. To calculate the total ΔGbind, the free solvation energy (polar and nonpolar solvation energies) and potential energy (electrostatic and van der Waals interactions) of each protein–ligand complex were analyzed. The MM-PBSA of all protein–ligand complexes was calculated for the last 30 ns time period. The average binding energy was then calculated using the “python” script included in g_mmpbsa.

## Results and Discussion

### Predictive Modeling and Virtual Screening

In this study, we built ten models with various hyperparameters, which were manually improved and examined by using statistical parameters (Table 1), and among all of them, the best model (number 5) was selected with a learning rate of 0.01, 30 epochs, 16 batch size, and two hidden layers. In this model, the neuron numbers were 512 and 100, the activation function was ReLU, the drop out was 0, and the output function was sigmoid.

**TABLE 1:** Manual optimization of hyperparameters to select the best deep learning model.

S. no	Model ID	Epoch	Hidden layers	No. of neurons	R2	Loss	MSE	RMSE	MAE
1	8RK310Z288WQJ5O4018J	80	3	1000,500,50	0.65	1.92	1.92	1.39	1.16
2	56002O1R7S0064Y9B4W0	60	3	1000,500,100	0.67	0.86	0.86	0.95	0.68
3	7A8NBRB65PRTM3U32822	80	3	50,200,100	0.62	0.84	0.84	0.91	0.69
4	1EUW57SF3HK089916UQ3	80	3	1500,1000,700	0.65	0.8	0.93	0.88	0.72
**5**	**H2QD4W391MZ025E3390D**	**30**	**2**	**512,100**	**0.67**	**0.78**	**0.78**	**0.79**	**0.68**
6	0RE2551MR14Q4V2593NO	30	3	1000,800,600	0.61	0.86	0.86	0.93	0.79
7	7OYBO5XRK1T5L3196TA2	30	2	128,512	0.6	0.89	0.89	0.94	0.72
8	LJM6WSWR3566O9Y4509X	30	2	1024,2048	0.65	0.9	0.9	0.96	0.69
9	7COSHT043Z10CE2J86S0	30	2	1024,512	0.66	1.03	1.03	1.01	0.8
10	X68C7XF4JQ533HR9XW10	30	2	1024,100	0.62	0.83	0.91	0.83	0.7

These bold values indicate the hyperparameters chosen for the deep learning model in this study.

**FIGURE 2:**
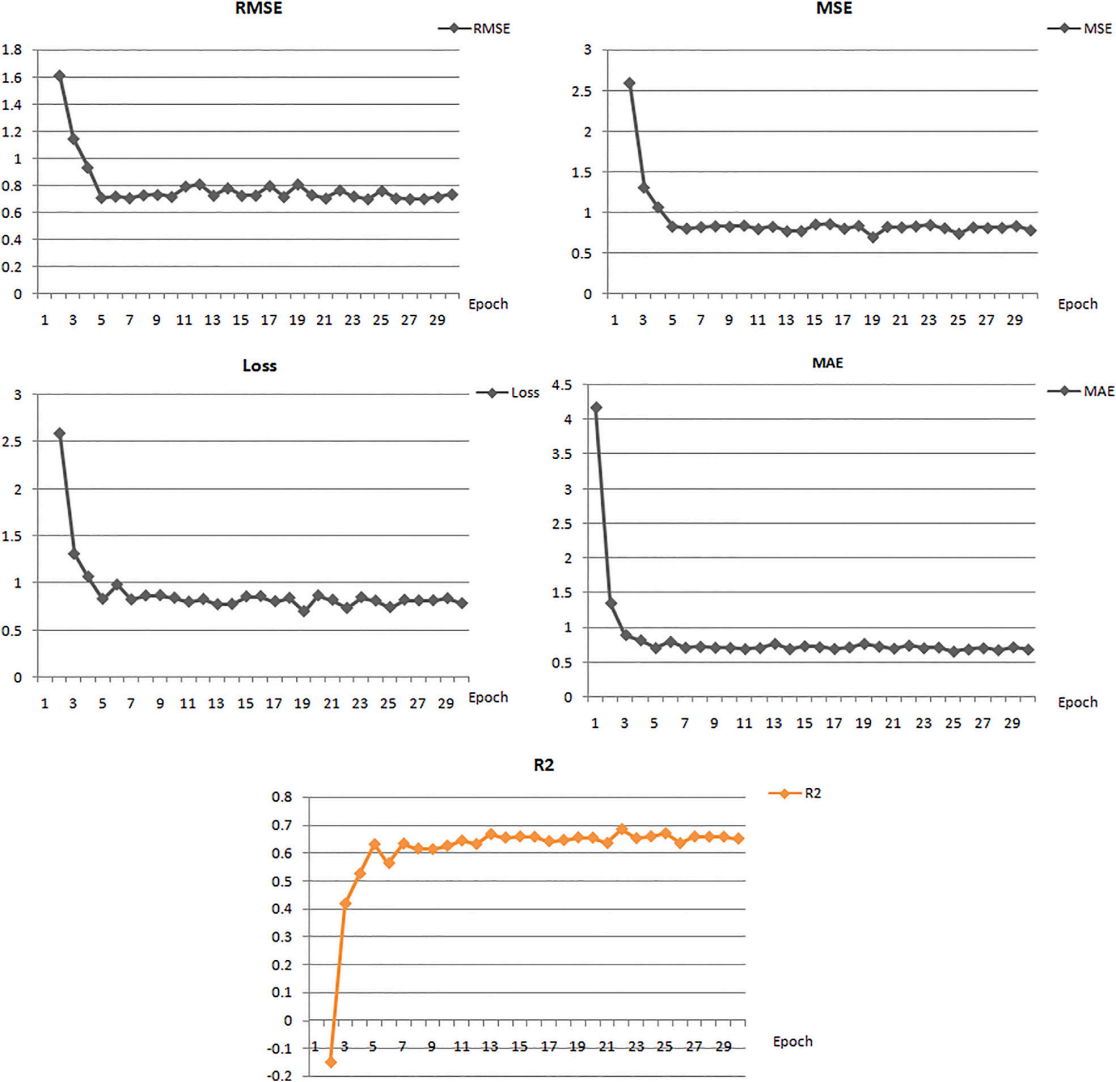
Performance of the best deep learning regression model for MMP-9 protein.

The best model exhibited a reasonable range of statistical parameters and displayed good results with a 0.78 loss value, a 0.67*R*2 value, 0.79 RMSE value, 0.78 MSE value, and a 0.68 MAE value (Figure 2). The deep learning model was then used to perform virtual screening on the dataset of the drug bank chemicals, which has a library of 9,102 compounds. The best predicted model screened 500 hits by virtual screening with deep-learning scores ranging from 11.7 to 3.4.

Furthermore, to obtain hits with a better score value than the reference inhibitor, the structure of the reference was submitted to the deep screening server and the score was predicted using the constructed model. The hits with the best scores were selected based on a comparison between the scores of the binding inhibitor of MMP-9 and all the 500 screened ligands. A total of 38 hits were found to have a score higher than the reference molecule (8.06) (Supplementary Figure S1). All the selected 38 compounds ranging from 11.7 to 8.06 were subjected to Lipinski’s rule of five predictions before molecular docking.

### Molecular Docking and Binding Mode Analysis

To predict the binding interactions of the screened compounds and the MMP-9 receptor, we used the molecular docking approach using AutodockVinaPyRx open-source software (GUI version 0.8). Before the screening, the docking protocol was validated by re-docking the reference molecule into the binding pocket of the active site of the MMP-9 protein. The result showed that the docked reference was completely superimposed with the co-crystallized reference molecule with an RMSD value of 1.2. Furthermore, the potential inhibitors of MMP-9 were identified by inspecting the docked poses of the ligands with the key active sites of MMP-9, such as the catalytic zinc ion, and amino acid residues of the S1_loop (pocket). The binding site of MMP-9 (PDB ID:1GKC) was evaluated by PDBsum (Laskowski et al., 2018), and Gly186, Leu187, Leu188, Ala189, Leu222, Val223, His401, Glu227, His405, Val398, His411, Leu243, Tyr423, Pro421, and Met422 were considered as binding site residues (Figure 1). The catalytic center is composed of the active-site zinc ion, coordinated by three histidine residues (401, 405, and 411) and the essential glutamic acid residue (402).

**FIGURE 3:**
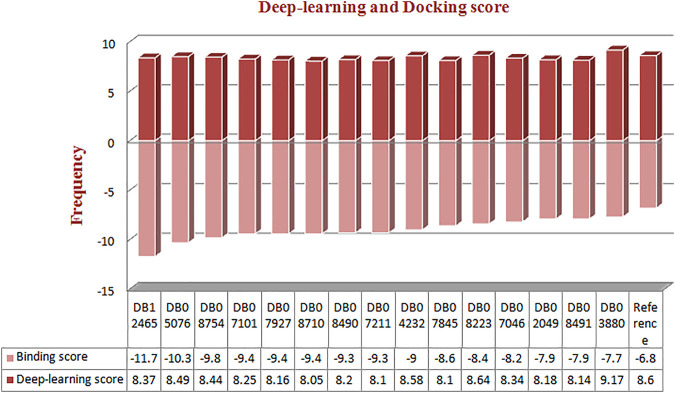
Frequency distribution graph of docked compounds over the range of deep learning and docking scores.

Molecular docking was performed for the 23 ligand molecules at the S1-pocket of the catalytic domain of MMP-9. All conformers of the ligands were ranked according to their docking score. The top 15 best docked conformers (Figure 3) with a higher affinity for the MMP-9 receptor than the reference were selected and subjected to an analysis of the binding mode and molecular interactions in the binding cavity of MMP-9. Furthermore, those compounds, involving binding to the catalytic zinc ion and the S1 pocket of MMP-9, were considered preferential in the interaction analysis. It can be seen from the interaction analysis results that the nine compounds do not show interactions with zinc (Supplementary Figure S2), so these compounds were not considered for further analysis (Figure 3).

Six compounds have been proven to exhibit an interaction with zinc and high activity against MMP-9. Therefore, these six compounds were considered for further study.

**FIGURE 4:**
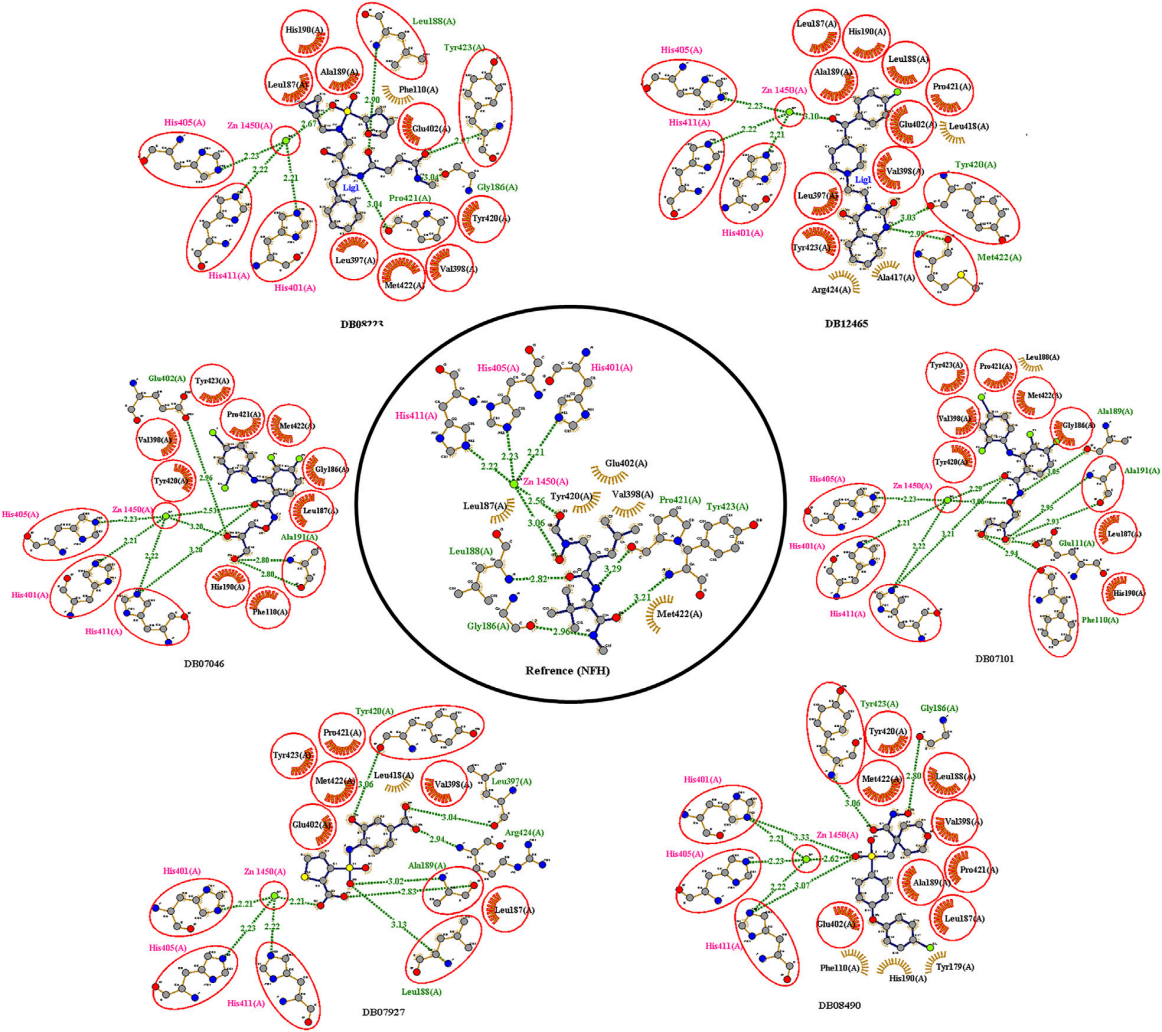
2D interactions of the top protein–ligand complex obtained by molecular docking.

The reference compound with binding energy −6.8 kcal/mol had formed a predominant hydrogen bond interaction with Leu188, Gly186, Tyr423, and Pro421 in addition to five hydrophobic bonds in the catalytic domain of MMP-9 (Figure 4).

Compound DB12465 showed the lowest binding energy −11.7 kcal/mol compared to all the compounds and exhibited interactions with Tyr420, and Met422 in the active site residues of the MMP-9 catalytic domain. We also found that it also established hydrophobic contacts with Glu402, Val398, Tyr423, Leu397, Pro421, Leu187, His190, and Ala189.

Compound DB07101 showed a good binding affinity (−9.4 kcal/mol) and formed four hydrogen bonds with Phe110, Ala191, Ala189, and Glu111 of the S1-pocket’s residues. However, DB07927 with binding energy (−9.4 kcal/mol) interacted *via* hydrogen bonding with Leu397, Arg424, Ala189, Leu188, and Tyr 420 by binding in the S1-pocket of MMP-9.

Compound DB08490 has also shown good binding energy (−9.4 kcal/mol) and formed two hydrogen bonds with Tyr423 and Gly186 in the MMP-9 catalytic domain. Ligand DB08223 was hydrogen-bonded to Leu188, Tyr423, Pro421, and Gly186, and established hydrophobic contacts with Leu397, Glu402, Tyr420, Val398, His190, Leu187, Met422, and Ala189 residues of the MMP-9 S1-pocket.

Compound DB07046 was hydrogen-bonded to Glu402 and Ala191, and had hydrophobic contacts with residues Tyr423, Pro421, Met422, Leu187, Gly186, Tyr423, His190, Val398, and Phe110 of the MMP-9 S1-pocket.

Furthermore, it is to be noted that all six compounds form H-bonds with Zn^2+^ and the coordination bond distances between Zn^2+^, and three histidines His405, His401, and His411 (His triad that holds Zn) are 2.23, 2.22, and 2.21, respectively, which is the same for all the ligands and reference molecule (Figure 4).

The present docking and interaction studies showed that all six compounds could inhibit the MMP-9 catalytic domain by binding at the S1-pocket and Zn^2+^ ion significantly similar to the reference with similar interactions at the same site. The result indicated that these hits may act as potent inhibitors for MMP-9.

### MD Simulation

The flexibility, structural behavior, and stability of the top six, highly active docked compounds were assessed by 100 ns MD simulation. It provides insights into the dynamic behavior of compounds, and the information obtained is often used in drug discovery. MD simulation was conducted for all the six protein–ligand complexes. To compare the results, the MD simulation was also performed on the MMP-9 protein without the ligand. Furthermore, the root mean square deviation (RMSD), root mean square fluctuations of the residues (RMSF), the radius of gyration (Rg), and other parameters were also examined for each system to ensure the conformational stability of the protein. After the RMSD analysis, out of six compounds, four compounds, with the IDs (DB07101, DB07927, DB08490, and DB12465) showed promising inhibitory activity against MMP-9 (3D structure of these four compounds is shown in Supplementary Figure S3). Therefore, only these four compounds were further analyzed and described.

### Root Mean Square Deviation and Fluctuations in the Residual Components

**FIGURE 5:**
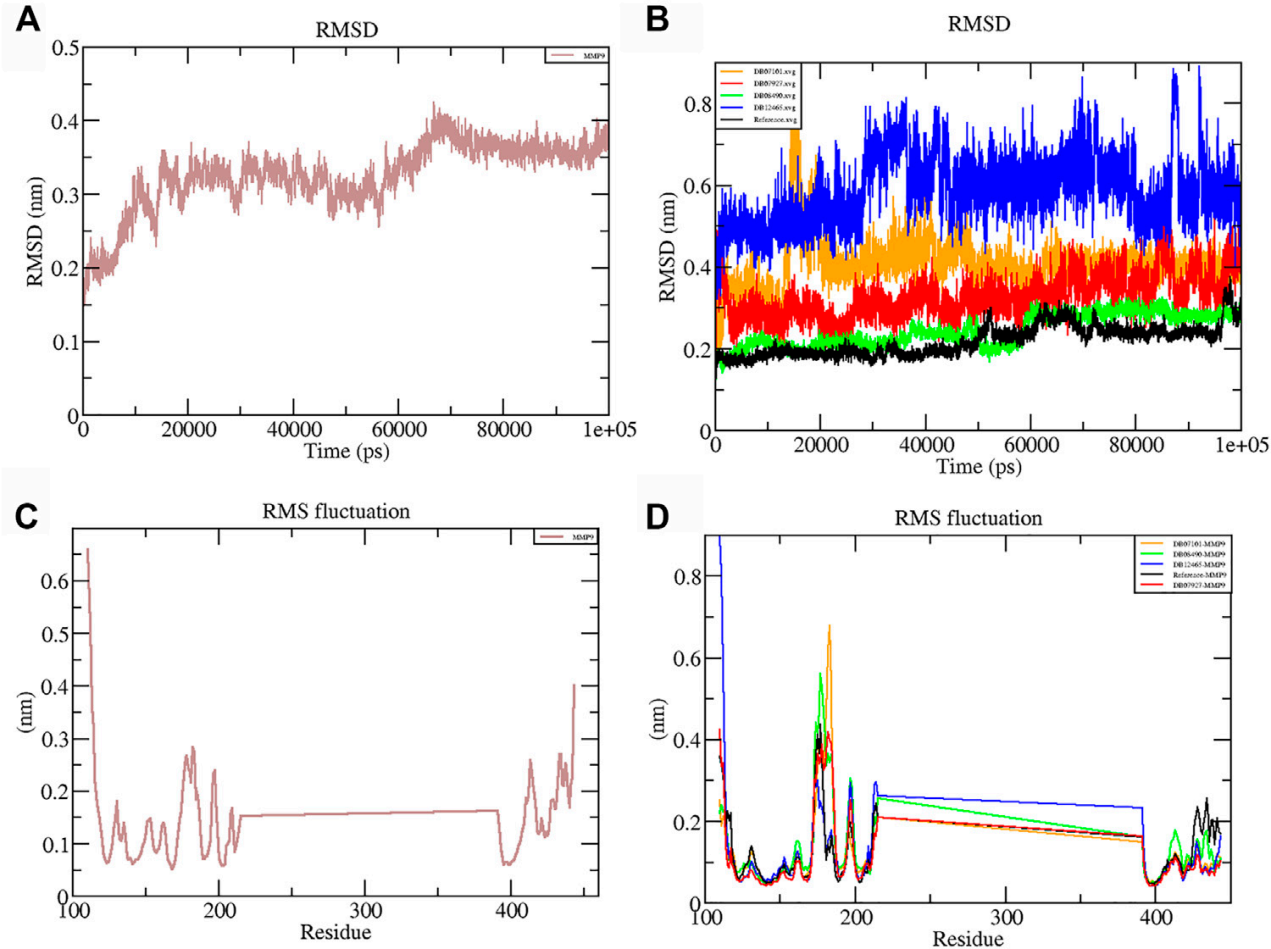
Graphs representing the RMSD plot of protein MMP-9 **(A)** and MMP-9–ligand complexes **(B)**; RMSF plot of MMP-9 **(C)** and MMP-9–ligand complexes **(D)**.

RMSD is a measure of the average distance between the backbone residues and atoms of a protein. The RMSD of MMP-9 was calculated against the four complexes; a graph was plotted using the three-dimensional Xmgrace plotting tool to compare the stability of the protein backbone and Cα atoms. The protein Cα and backbone residues showed minimal fluctuations before equilibration of the system and were found to be stable throughout the MD simulation study. As seen in Figure 5, all complex systems reached a steady state very quickly and remained stable until the end of the simulation time, whereas the native protein MMP-9 stabilized later with an increasing RMSD value (Figure 5A). One possible explanation is that the MMP-9 protein is not restricted, and can therefore modify its conformation to achieve a more stable geometry.

The graph showed that the maximum RMSD value, which belonged to the complex DB12465–MMP-9, was approximately 0.58 nm, and it was higher than those of other complex systems. But the complex had a fluctuation of less than 0.2 nm, which is completely acceptable. Other complexes DB07927–MMP-9 and DB08490–MMP-9 showed good interaction with the protein active site similar to the reference molecule (0.21 nm). In both cases, the average RMSD values were calculated as 0.32 and 0.24 nm, respectively. It decreased because of the interactions between the protein residues and the ligands which limited conformational changes in the protein structure. Therefore, from the RMSD profile, it can be seen that all the four compounds, after reaching an equilibration state, fit in the active site and stabilized during the MD simulation (Figure 5B).

The root mean square fluctuation (RMSF) of the backbone residues of all the four complexes was plotted against the residue numbers to undertake a deeper investigation into the variations in protein flexibility during the simulation. As depicted in Figure 5C, the residues of the active site such as Gly186, Leu187, Leu188, Ala189, Leu222, Val223, His401, Glu227, His405, His411, Leu243, Tyr423, Pro421, and Met422 had small fluctuations in comparison with their adjacent residues in both the complexes. However, in the case of all ligands, a tiny peak was observed around the residue 170–178, which was enriched in hydrophobic residues and not involved in ligand interaction (Figure 5D). But the fluctuation was less than 0.2 nm, which is completely acceptable.

Overall, protein residual fluctuations in all complexes were found to be minimal (Table 2). From the result, we can depict that all the four ligands interacted through the active site residues of MMP-9 and that the protein residues involved in the active site of all the complexes remained stable during the entire MDS run. This result of both the RMSD and RMSF demonstrated that the selected ligands were potential inhibitors of MMP-9.

**TABLE 2:** Active site residues and their RMSF values (angstrom).

Protein–ligand complex	Hydrogen bond interaction			Hydrophobic bond interaction		
	No. of bonds	Residues involved	RMSF value	No. of bonds	Residues involved	RMSF value
DB07101–MMP-9	7	Ala189	0.08	8	Tyr423	0.09
					Val398	0.05
		Ala191	0.08		Tyr420	0.09
		Glu111	0.19		His190	0.08
		Phe110	0.15		Pro421	0.10
		His401	0.05		Met422	0.10
		His405	0.07		Gly186	0.19
		His411	0.10		Leu187	0.11
DB08490–MMP-9	5	Gly186	0.12	8	Val398	0.05
		Tyr423	0.09		Ala189	0.08
		His401	0.05		Val398	0.05
		His405	0.07		Leu188	0.09
		His411	0.15		Tyr420	0.09
					Glu402	0.06
					Leu187	0.11
					Pro421	0.11
DB07927–MMP-9	8	Tyr420	0.07	6	Val398	0.04
		Leu397	0.04		Pro421	0.09
		Ala189	0.06		Tyr423	0.08
		Arg424	0.08		Glu402	0.05
		Leu188	0.07		Leu187	0.15
		His401	0.04		Met422	0.09
		His405	0.06			
		His411	0.10			
DB12465–MMP-9	5	Tyr420	0.07	9	Leu187	0.09
		Met422	0.09		Ala189	0.06
		His401	0.04		His190	0.06
		His405	0.07		Leu188	0.10
		His411	0.09		Pro421	0.08
					Glu402	0.05
					Val398	0.04
					Leu397	0.04
					Tyr423	0.08
Reference–MMP-9	7	Leu188	0.05	5	Glu402	0.05
		Gly186	0.10		Tyr420	0.07
		Tyr423	0.08		Val398	0.04
		Pro421	0.08		Met422	0.08
		His401	0.05		Leu187	0.06
		His405	0.07			
		His411	0.10			

### The Radius of Gyration and Hydrogen Bond Analysis

**FIGURE 6:**
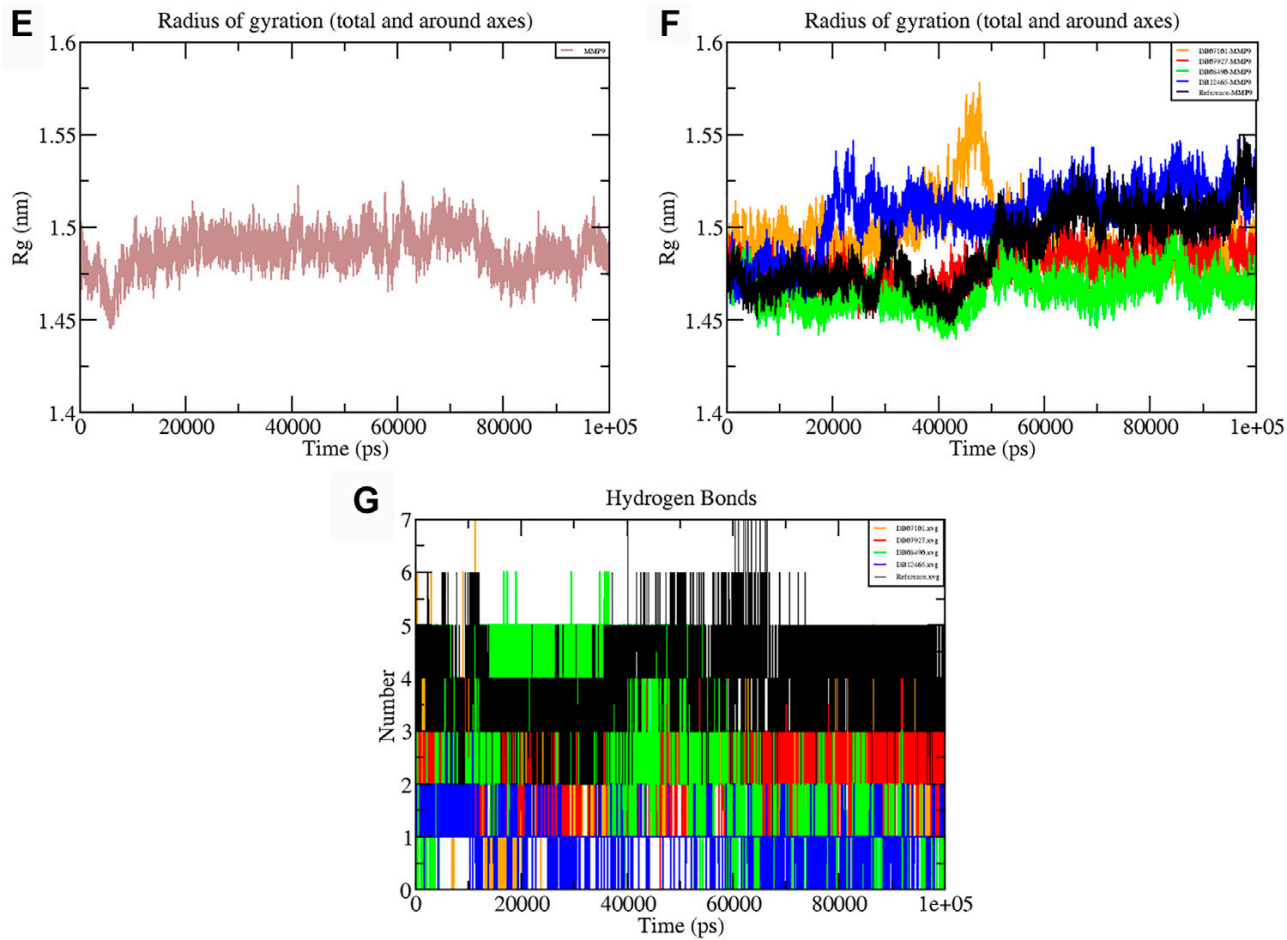
Graphs representing the Rg of **(E)** MMP-9 **(F)** MMP-9–ligand complexes **(G)** H-bonds during the 100 ns simulation period.

The Rg shows the compaction level in the protein structure during the simulation. As seen in Table 3, the average Rg values calculated for all the four complexes and native protein are approximately the same as the reference molecule, and the continuity of all complexes is retained. All interacting residues show the Rg in the range of 0.12 nm, indicating minimal fluctuations and greater stability throughout the MD simulation study. Therefore, from the graph (Figures 6E, F) of the Rg, we can demonstrate that the binding of ligands does not change the folding behavior of the protein, and that it was maintained during the MDS run.

**TABLE 3:** Average values of different parameters, RMSD, Rg, H-bonds, and interaction energy.

Complex	Average RMSD (nm)	Average Rg (nm)	H-bonds	Interaction energy (kJ/mol)
Native protein (MMP-9)	0.32 ± 0.03	1.2 ± 0.01	—	—
DB07101–MMP-9	0.40 ± 0.04	1.2 ± 0.008	07	−131.352
DB07927–MMP-9	0.32 ± 0.04	1.2 ± 0.01	04	−147.098
DB08490–MMP-9	0.24 ± 0.03	1.2 ± 0.01	06	−110.611
DB12465–MMP-9	0.58 ± 0.06	1.2 ± 0.01	06	−171.081
Reference–MMP-9	0.21 ± 0.03	1.2 ± 0.009	07	−135.604

The hydrogen bond formation/deformation indicates the number of H-bonds formed or broken during the MD simulation. For the reference molecule, almost five hydrogen bonds were found to be constant throughout the simulation. In contrast, an extra two hydrogen bonds appeared from 40 to 75 ns (Figure 6G). Complex DB08490–MMP-9 displayed five constant hydrogen bonds, whereas complex DB07927–MMP-9 showed three H-bonds during the MDS run, although an additional H-bond was also observed during the 100 ns simulation period. Similarly, around three hydrogen bonds were calculated for complex DB07927–MMP-9 and seven for DB07101–MMP-9. Only four H-bonds were found to be constant in the case of complex DB07101–MMP-9 during MDS run. However, during the period of 100 ns, most hydrogen bond numbers remained constant, suggesting the molecular or structural stability of all compounds with MMP-9.

### Interaction Energy Analysis

**FIGURE 7:**
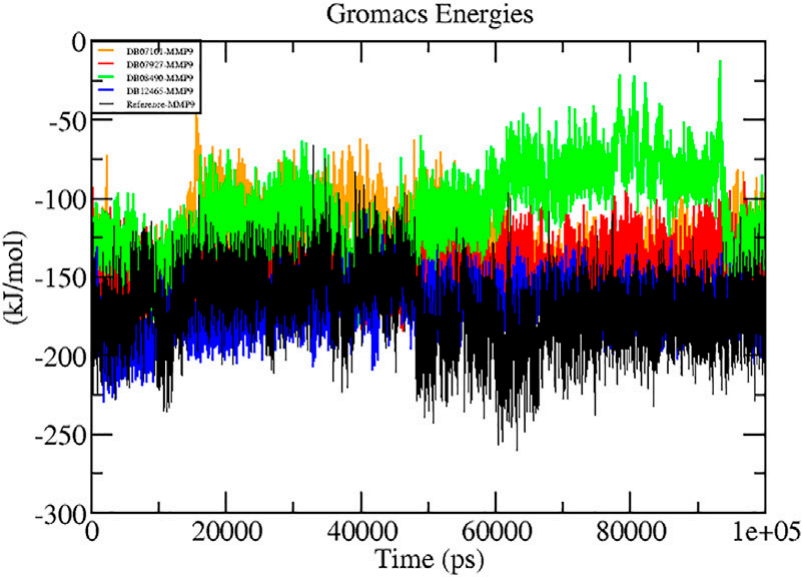
Graphs representing the interaction energy of protein–ligand complexes.

A thorough study was performed to quantify the free energy of interactions between protein–ligand complexes using the Parrinello–Rahman parameter implemented in GROMACS to confirm the binding score produced by molecular docking experiments. The average interaction energy of all the complexes was observed in the acceptable range of −100–200 kJ/mol. In the 100 ns simulation period, the highest interaction energy of complex DB12465–MMP-9 was found to be −171.081 kJ/mol followed by complex DB07927–MMP-9 which was −147.098 kJ/mol. The energy of both complexes was found to be significantly higher than that of the reference molecule (−135.604 kJ/mol) (see Table 3). In addition, all other complexes DB07101–MMP-9 and DB08490–MMP-9 also showed good interaction energies of −131.352 kJ/mol and −110.611 kJ/mol, respectively. Therefore, the average interaction energy of all the complexes was observed to be in the acceptable range over the 100 ns simulation period (Figure 7). It confirmed the molecular docking results and suggested that these compounds had a good affinity for MMP-9.

### Principal Component Analysis and Gibbs Free Energy Analysis

Typically, only the first few eigenvectors define the overall motion of the protein subspace, where most of the protein dynamics occur (Yang et al., 2014). Therefore, to identify important eigenvectors responsible for the overall motion and dynamics, PCA was used to investigate the overall motion and essential dynamics of all protein–ligand complexes.

**FIGURE 8:**
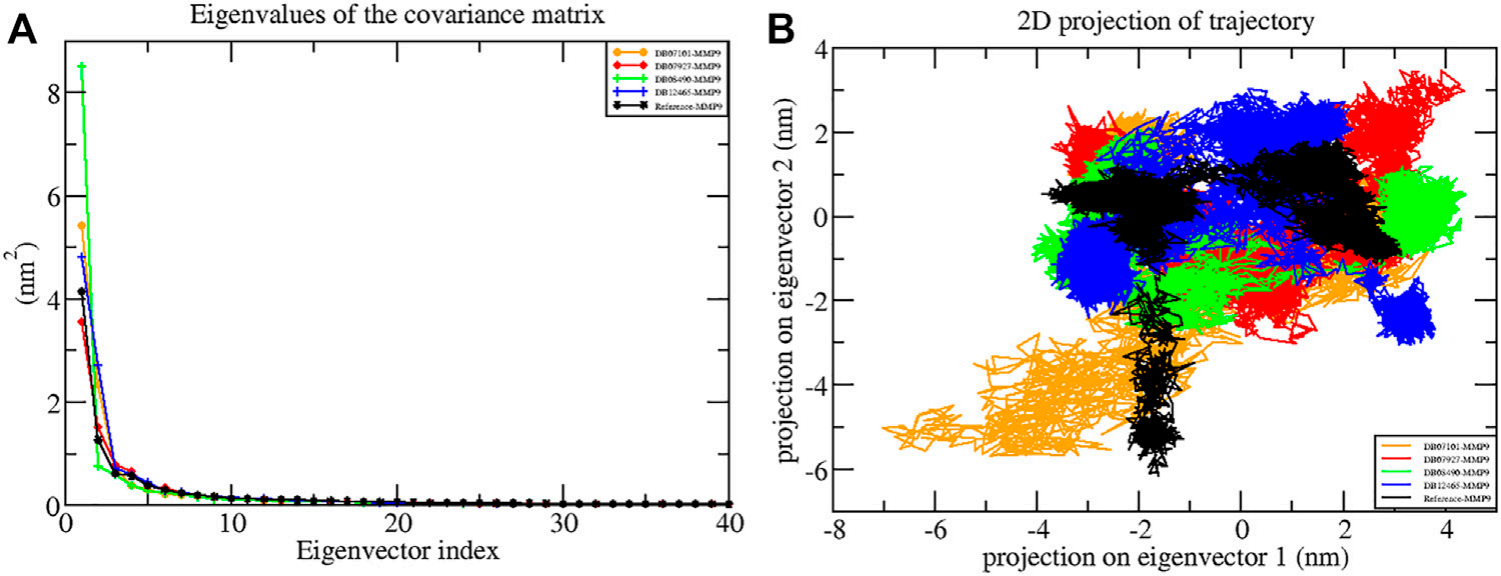
Principal component analysis showing **(A)** plots of eigenvalues vs first 40 eigenvectors and **(B)** 2D projection plots during the 100 ns simulation period.

In the present study, the first 40 eigenvectors were used to calculate the total motion in each case. The graph of the eigenvalues (Figure 8A) was generated after plotting the eigenvalues against the eigenvectors. From the calculation, the motions for the first ten eigenvectors were accounted to be 78% for DB07101–MMP-9, 79% for DB07927–MMP-9, 82% for DB08490–MMP-9, and 81% for DB12465–MMP-9 complexes during the 100 ns simulation period. Therefore, the result indicates that all the complexes showed very few motions similar to the reference compound (79%).

Another way to achieve complex dynamics is to use PCA to generate 2D projection plots. A 2D projection of the trajectories of all the complexes in the phase space for the first two principal components, PC1 and PC2, was developed, as is shown in Figure 8B. The complex occupying less phase space has a more stable cluster, whereas the complex occupying more space shows a less stable cluster. From the figure, it can be observed that all four complexes are highly stable as they occupy less space in the phase space as the reference molecule.

**FIGURE 9:**
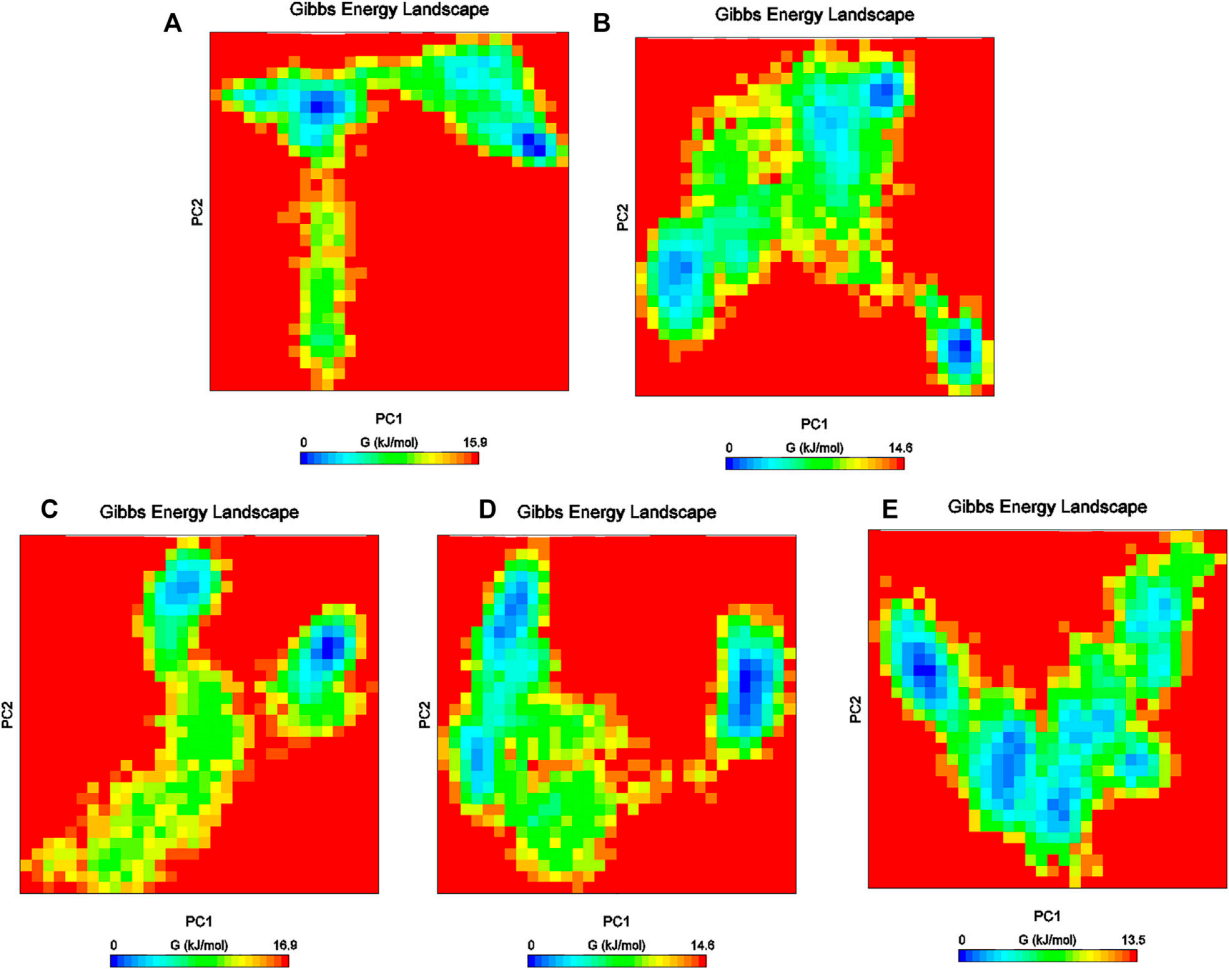
Gibbs energy plot of **(A)** reference–MMP-9, **(B)** DB12465–MMP-9, **(C)** DB07101–MMP-9, **(D)** DB08490–MMP-9, and **(E)** DB07927–MMP-9 complex.

Furthermore, the Gibbs free energy landscapes were obtained from the PC1 and PC2 coordinates, and are shown in Figure 9. In these plots, ΔG values ranged from 0 to 15.9 kJ/mol, 0 to 16.9 kJ/mol, 0 to 13.5 kJ/mol, 0 to 14.5 kJ/mol, and 0 to 14.0 kJ/mol for (reference-MMP-9) complexes DB07101–MMP-9, DB07927–MMP-9, , DB08490–MMP-9, and DB12465–MMP-9, respectively. All the complexes represent significantly similar energy as the reference except the DB07927–MMP-9 complex, which was slightly low. The result indicates that these compounds follow the energetically favorable transitions during the dynamics simulation (Figure 9).

### Binding Free Energy Calculations

The complex stability is further assessed by calculating the binding free energy of top compounds (last 30 ns) using the g_mmpbsa tool (Kumari et al., 2014). From the results obtained from the MM-PBSA analysis, it was concluded that the ΔG_Bind_ of the DB12465-MMP-9 complex depicted the highest binding affinity or lowest binding free energy of −115.669 KJ/mol.

The resulting ΔG_Bind_ of other complexes DB08490–MMP-9 and DB07101–MMP-9 were found to be −87.148 KJ/mol and −77.342 KJ/mol, respectively, which were better than the reference molecule (−73.082 KJ/mol). Complex DB07927–MMP-9 showed a significantly similar binding affinity with a ΔG_Bind_ value of −62.234 KJ/mol. The details of MM-PBSA calculation for the top four complexes are summarized in Table 4. From the table, it can be seen that van der Waals interactions play an important role in the simulation of all complexes, contributing significantly more to the total interaction energy than the other energies. The result indicates that all four compounds bind effectively to the active site of the MMP-9 protein and could be used as a potential drug candidate.

**TABLE 4:** Table representing the van der Waal, electrostatic, polar salvation, SASA, and binding energy for protein–ligand complexes.

Protein–ligand complex	van der Waal energy (KJ/mol)	Electrostatic energy (KJ/mol)	Polar solvation energy (KJ/mol)	SASA energy (KJ/mol)	Binding energy (KJ/mol)
DB07101–MMP-9	−157.240±15.654	−60.629±11.409	158.076±22.426	−17.550±1.576	−77.342±15.435
DB08490–MMP-9	−123.624±30.165	−26.038±19.213	65.191±19.076	−13.913±2.193	−87.148±22.175
DB07927–MMP-9	−153.550±13.097	−61.694±28.570	167.770±26.449	−14.760±0.999	−62.234±14.980
DB12465–MMP-9	−184.774±11.790	−16.571±8.639	102.641±15.507	−16.965±0.997	−115.669±14.466
Reference–MMP-9	−155.490±11.506	−62.844±10.331	162.839±9.967	−17.588±0.799	−73.082±10.629

## Discussion

MMP-9 has been linked to cancer pathophysiology as it plays an important role in ECM remodeling and membrane protein cleavage. MMP-9 degrades the basement membrane, as the basement membrane contains collagen, including type IV collagen, which can be degraded by MMP-9 (Hou et al., 2014). During tumor development, the destruction of the basement membrane is usually a critical step that supports tumor invasion and metastasis. MMP-9 expression can be unusually high in most cancers, and it is regulated in a complicated way. Indeed, in cancer patients, a high MMP-9 level is linked to a poor prognosis. MMP inhibitors can lower the invasiveness and migratory potential of tumor cells (Winer et al., 2018). MMP inhibitors that are specifically intended to be safe and effective are still a major topic in cancer research. Targeting Zn^2+^ and catalytic active sites, which are essential for protein activity, may block the enzymatic action of MMP-9. The computational technique evaluates the binding affinity and inhibitory potential of compounds against proteins, and therefore helps in the development of novel drug candidates.

**FIGURE 10:**
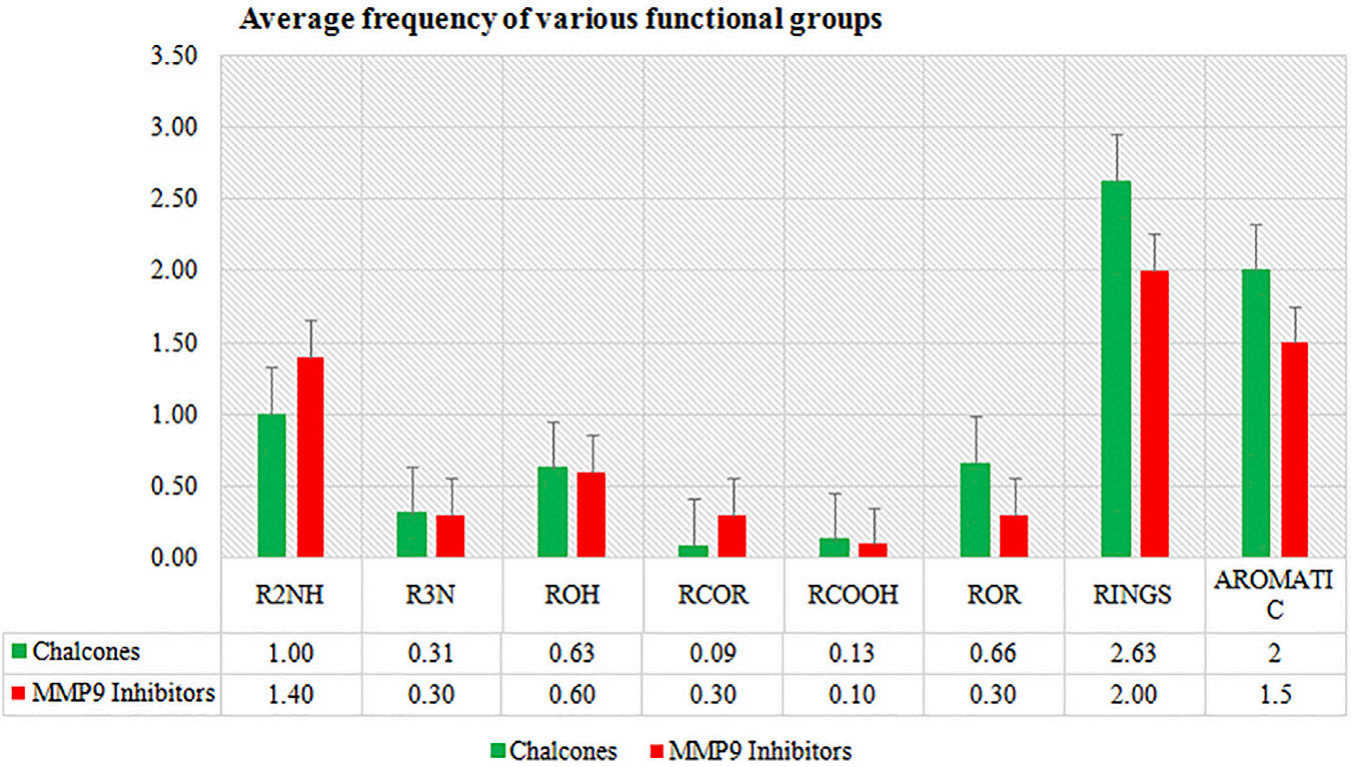
Average frequency of functional groups of MMP-9 inhibitors and top four compounds.

In the present study, *in silico* techniques have been used to investigate potential MMP-9 inhibitors. The screening of drug bank compounds was started with a deep learning model. Furthermore, the best model with good *R*2, RMSE, MSE, and MAE values was selected for screening. Prescreening by deep learning resulted in 500 compounds which were narrowed down to 23 drugs. The output was used as the input for molecular docking, resulting in a more reliable prediction. The docking result demonstrated that six compounds showed strong binding potential with MMP-9 and also had promising interactions with active site residues and Zn^2+^. All the compounds had a higher binding affinity toward MMP-9 than the reference molecule. Furthermore, during the MD simulation of 100 ns, only four hits showed good RMSD and RMSF values that reflect very tightly with MMP-9. The frequency estimation of the functional group of all four compounds was also calculated by R (version 3.4.3) software using ChemmineR “library.” Hit compounds were examined for eight functional groups: carbonyl group (RCoR), nitrile (RCN), primary amine (RNH2), carboxyl group (RCOOH), hydroxyl group (ROH), ether group (ROR), secondary amines (R2NH), and tertiary amines (R3N), as well as aromatic groups and rings. All of these were then compared with the inhibitors of MMP-9 (Figure 10). We observed that the rings and all other groups are present in both the MMP-9 inhibitors and screened compounds. Thus, these compounds are significantly similar and may have similar biological activities.

The first screened hit compound was Ketanserin (ID-DB12465). Ketanserin is a member of the class quinazolines and is an antagonist of the 5-HT receptor, which is used clinically for conditions including high blood pressure, anxiety, depression, and psychosis (Brogden and Sorkin, 1990). It has been reported that the inhibition of 5HT2B receptors leads to the death of HCC cells *in vitro* and reduces tumor growth in mice (Mansfield,). The compound also has a binding potential with MMP-9 and was found to have hydrogen bonds with the catalytic residue Glu-402 and His triad that holds Zn^2+^.

Mirdametinib (PD-0325901) (ID DB07101), the second screened hit compound, is an orally delivered, highly selective inhibitor of both MEK isoforms, MEK1 and MEK2, which prevents the activation and phosphorylation of mitogen-activated protein kinase (MAPK). Mirdametinib (PD-0325901) has already been shown to suppress pERK (Haura et al., 2010). In previous studies, Mirdametinib showed growth-inhibitory and antiangiogenic effects on glioblastoma, non-small-cell lung cancer (NSCLC), and melanoma tumor progression by inhibiting mitogen-activated protein kinases (MAPKs) (Henderson et al., 2010). The compound was found to have hydrogen bonds with S1-pocket’s residues of MMP-9, and it also regulates the catalytic activity by binding with Zn^2+^.

The third investigated compound CTS-1027 (ID-DB08490) has already been reported against MMP as a potential hepato-protective agent. It is a diphenyl ether, which is a type of organic compound. These are aromatic compounds that include two benzene rings connected by an ether group. It is a particularly potent inhibitor of human MMPs 2, 3, 8, 9, 12, 13, and 14 but not 1 or 7 (Kahraman et al., 2009). In our docking study, also showed binding with the S1-pocket and Zn^2+^ by forming H-bonds and hydrophobic interactions.

The fourth hit compound 3-{[(4-CARBOXY-2-HYDROXYANILINE]SULFONYL}THIOPHENE-2-CARBOXYLIC ACID ( ID-DB07927) belongs to the class of organic compounds known as sulfanilides. The compound also showed H-bonds with Tyr423, Gly186, and Zn^2+^ in the MMP-9 catalytic domain, which is critical for inhibiting the catalytic activity of the enzyme.

**FIGURE 11:**
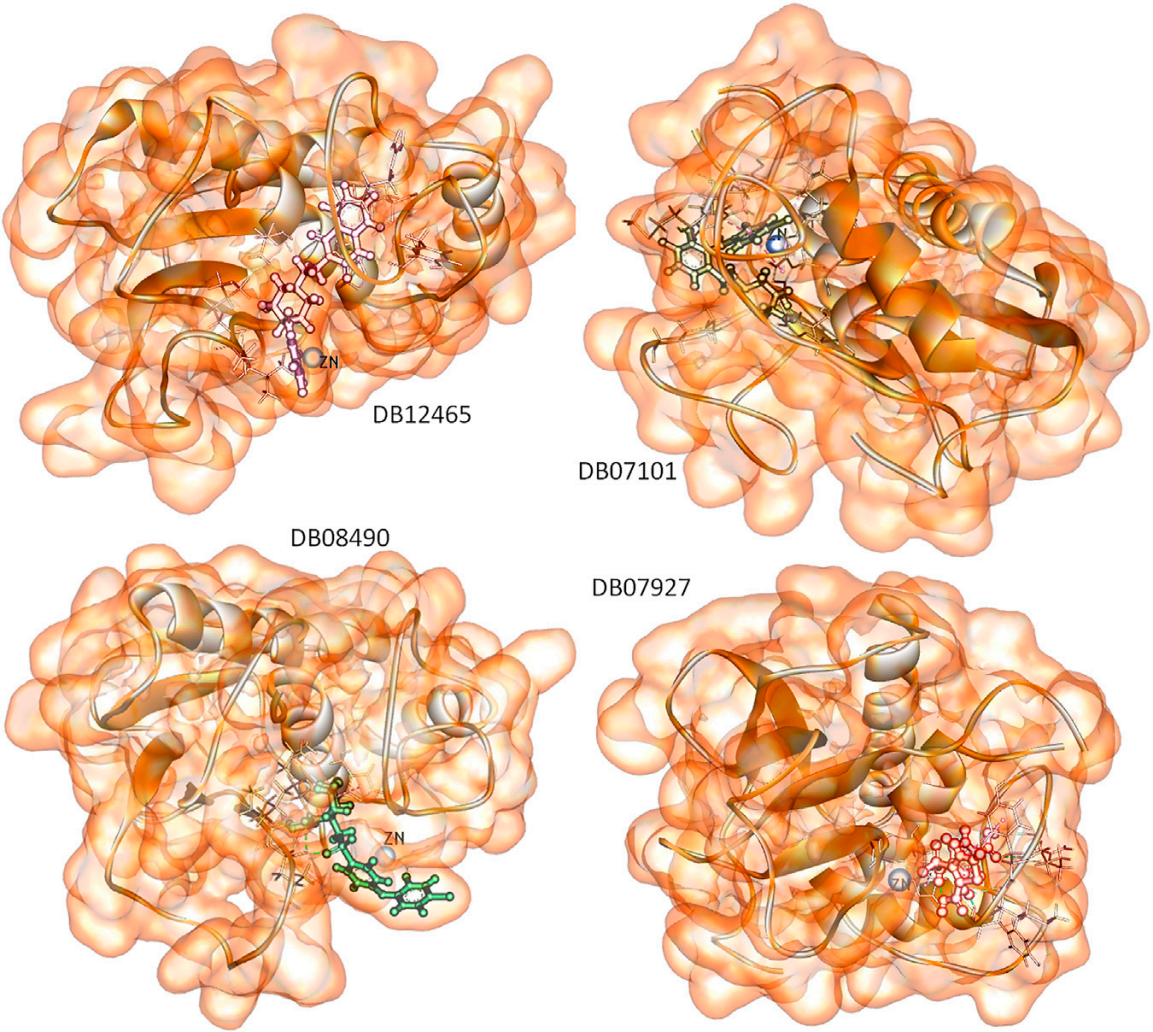
Snapshots of selected four ligands over the course of 100 ns MD simulation.

The MD simulation result suggests that all these four ligands interacted through the active site residues of MMP-9 and were stabilized in the active site region with very little fluctuation. The Rg result indicates the compactness of proteins and constant stability of all protein–ligand complexes throughout the MD simulation study. Furthermore, the continuous contribution of hydrogen bonding interactions to the binding pose analysis indicates that all compounds have stable interactions with MMP-9 proteins. The calculation of the interaction energy indicates the strength of protein–ligand complex systems. This study showed that all compounds have higher and significantly better interaction energy with proteins than reference compounds, and show an acceptable range of interaction energies. Finally, to more accurately estimate the binding preference of the selected ligand for MMP-9, we have examined the calculated binding free energies of all four complexes using the MM-PBSA method. All four compounds were high potential binders according to the calculated binding free energy. Furthermore, in Figure 11, we displayed the snapshots extracted from the trajectory of the MD simulations. It can be seen that all the four ligands are well accommodated with the MMP-9 pocket and ZN^2+^, which means that the protein–ligand complexes were associated during the 100 ns simulation period, and all of them have good binding with MMP-9.

We may conclude that our drug screening pipeline performed well due to the high efficiency of the deep learning approach and the relative accuracy of MD simulations.

The present study proved valuable in cancer prevention. Additionally, all four compounds have been reported to be active against cancer and other diseases, indicating strong possibilities that they could be potential hit inhibitors against MMP-9. The results of this study are useful for drug development.

## Conclusion

To obtain potent MMP-9 inhibitors, we used the deep learning-based method followed by docking to do preliminary screening to identify compounds that simultaneously target Zn^2+^ and the binding region of MMP-9 enzymes. Deep learning and molecular docking resulted in six hits that could inhibit MMP-9. Hence, using MD simulations, we explored the stability and binding process of the most interesting four ligands by analyzing RMSD, RMSF, H-bonds, ROG, interaction profile, and PCA. The results confirmed the high stability of these four hits in the active site of MMP-9. Interestingly, these four compounds are already being used for diseases and one compound is known to be an MMP-9 inhibitor. The overall results suggest that these hits may also be useful as lead molecules for designing more potent drugs to treat cancer or to act as suitable drug candidates against MMP-9.

## Data Availability

The original contributions presented in the study are included in the article/Supplementary Material, further inquiries can be directed to the corresponding author.
