# Supplementary material for: Identification of Zinc-Binding Inhibitors of Matrix Metalloproteinase-9 to Prevent Cancer Through Deep Learning and Molecular Dynamics Simulation Approach
Source: Front Mol Biosci. 2022 Mar 31;9:857430. doi: 10.3389/fmolb.2022.857430 (PMC9024349; doi:10.3389/fmolb.2022.857430)
Supplement: Supplementary file 1 [file DataSheet1.docx]

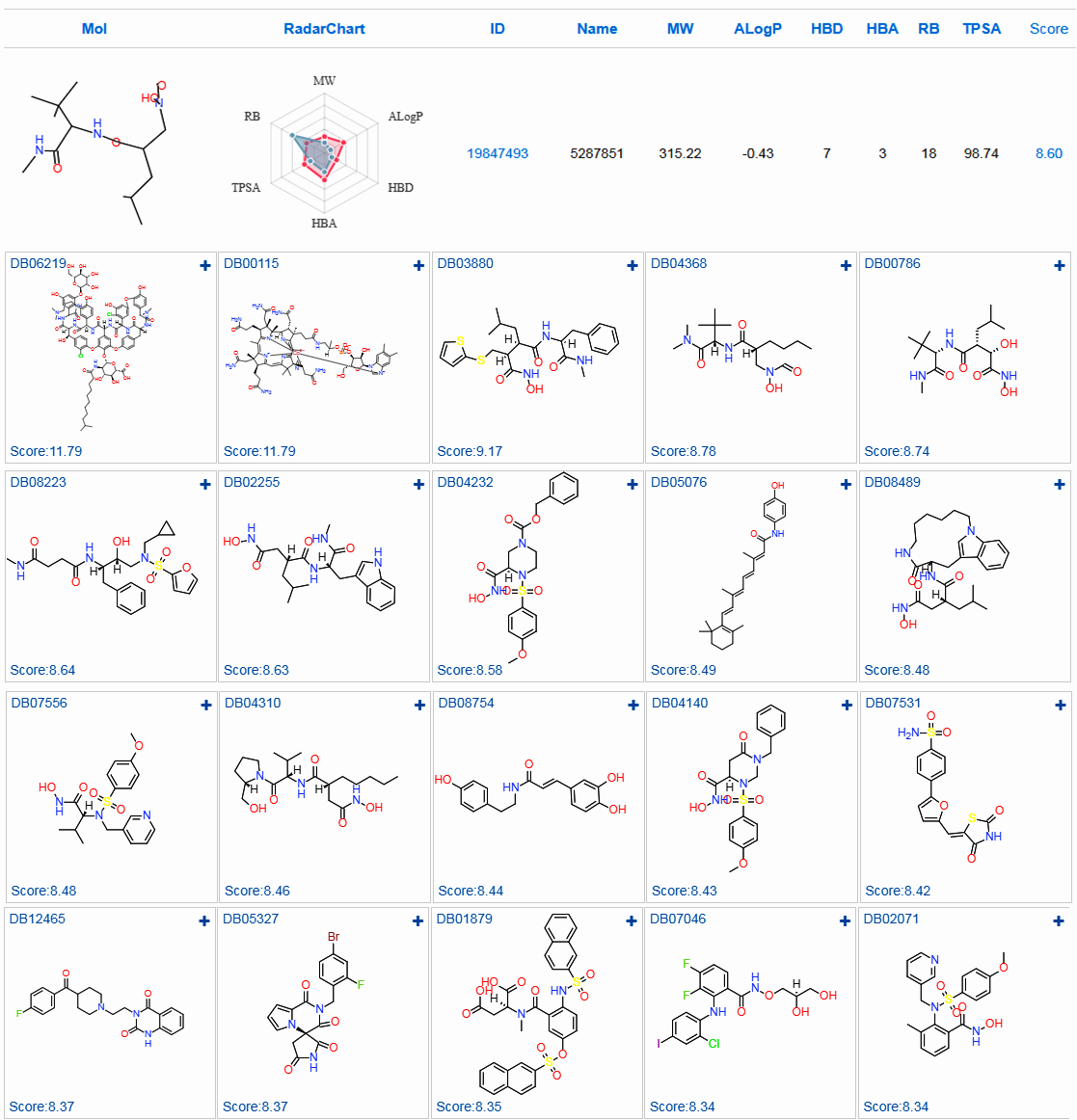


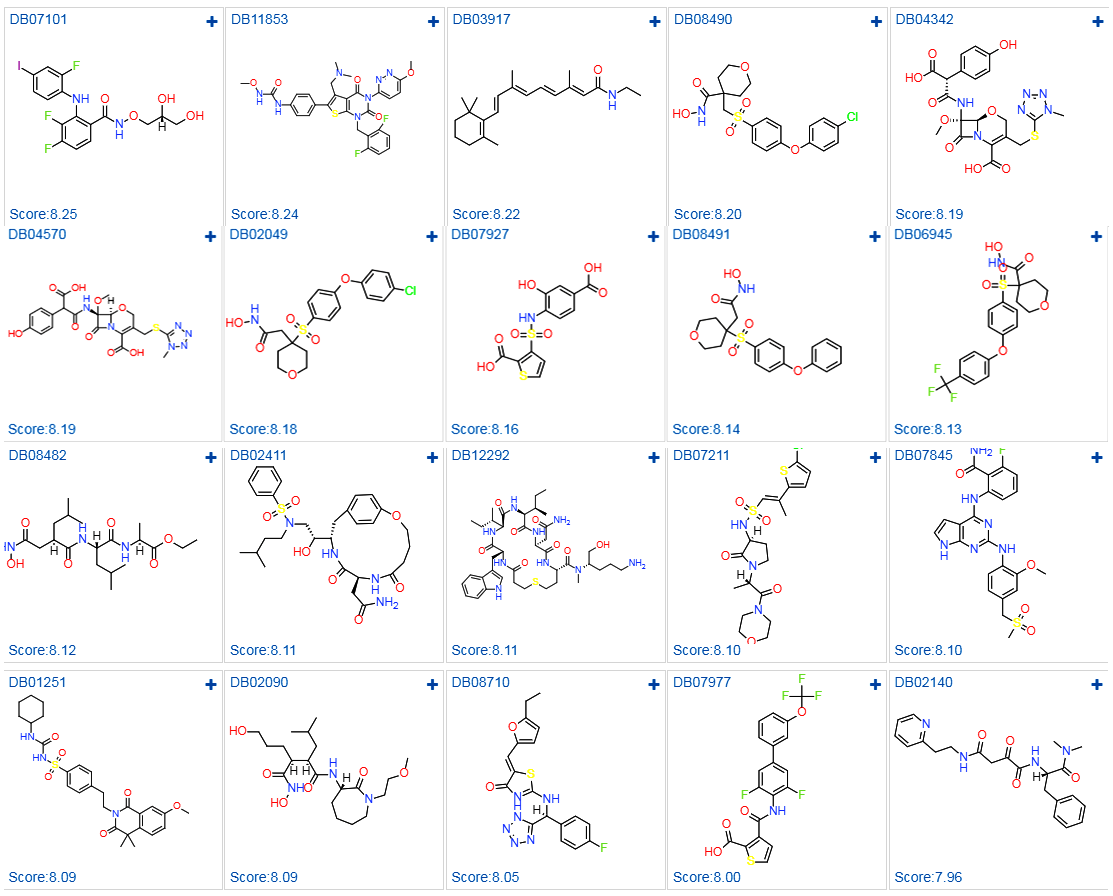


**Supplementary figure 1-** Total hit compounds screened by deeplearning method


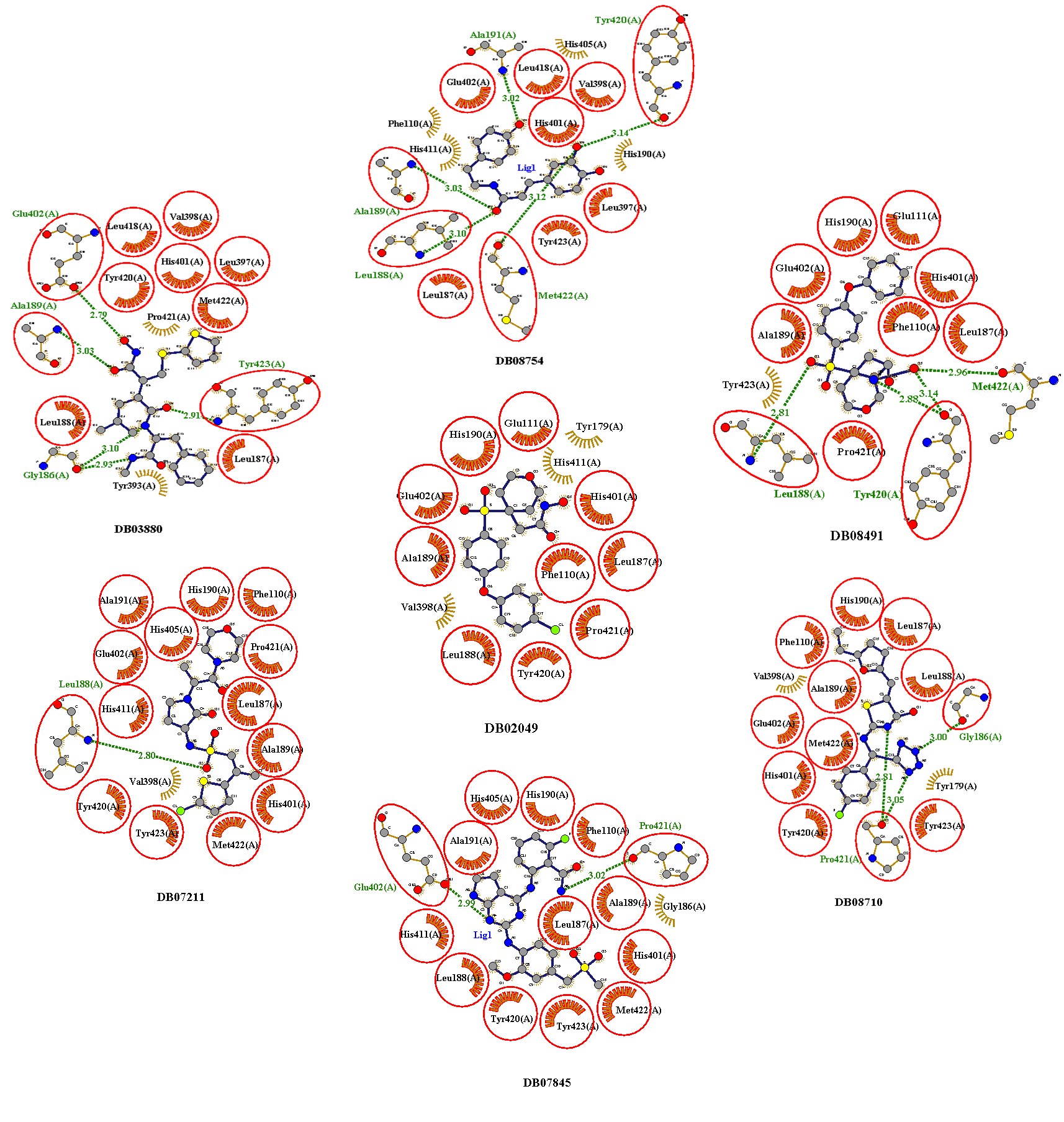


**Supplementary figure 2-** 2D plots of top docked compounds not showing interaction with zinc


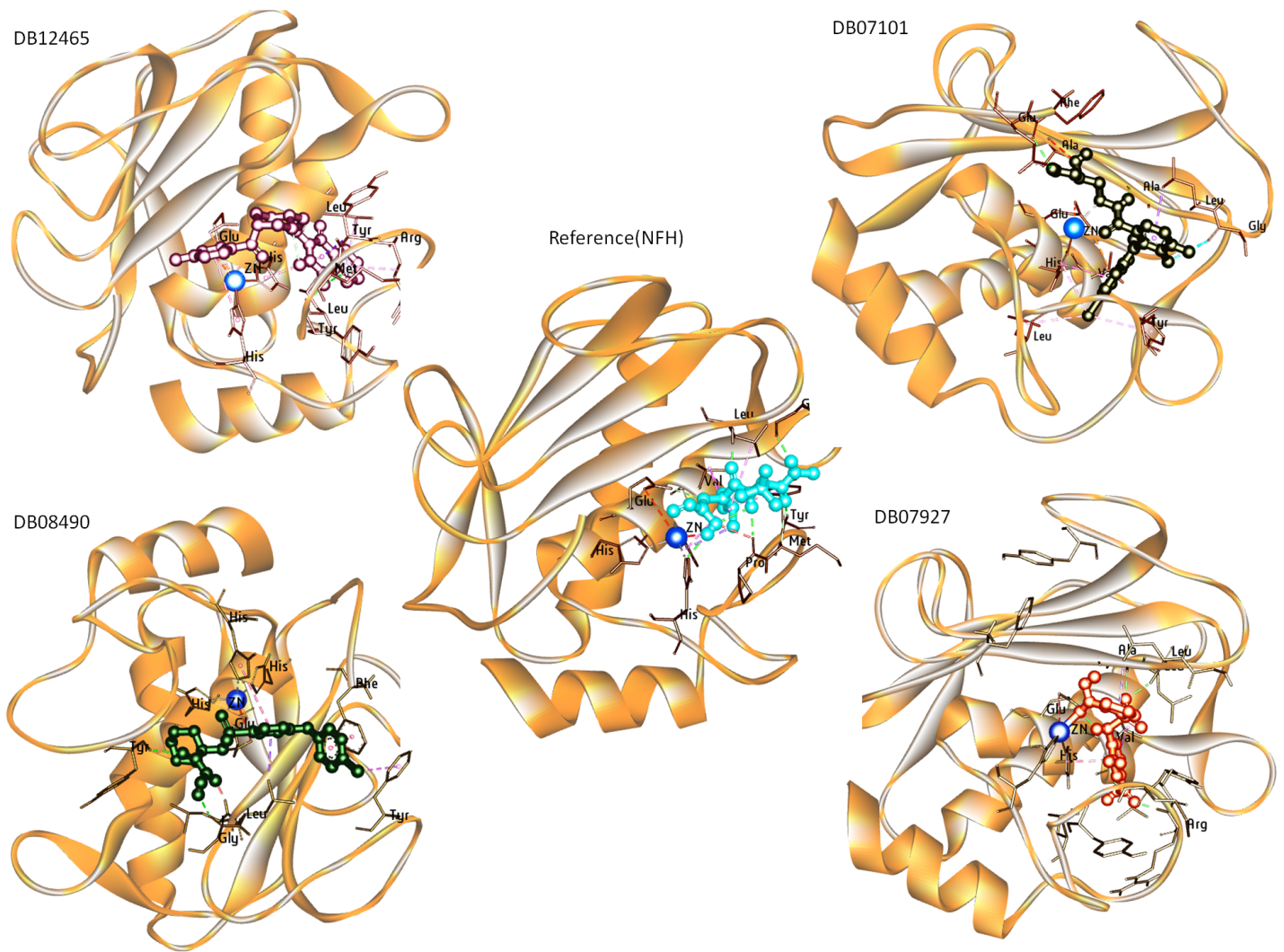


**Supplementary figure 3-** 3D plots of best four compounds and reference molecule after the 100 ns MD simulation
